# Effects of a 105 hours psychological training program on attitudes, communication skills and occupational stress in oncology: a randomised study

**DOI:** 10.1038/sj.bjc.6601459

**Published:** 2004-01-06

**Authors:** N Delvaux, D Razavi, S Marchal, A Brédart, C Farvacques, J-L Slachmuylder

**Affiliations:** 1 Service de Psychologie, Hôpital Erasme, Université Libre de Bruxelles, Route de Lennik 808, Bruxelles 1070, Belgium; 2 Psychosomatic and Psycho-oncology Research Unit, Faculté des Sciences Psychologiques et de l'Education, Université Libre de Bruxelles, Avenue A. Depage 30, 1050 Bruxelles et Institut Jules Bordet, Rue Héger Bordet 1, Bruxelles 1000, Belgium; 3 CAM, Groupe de Recherche et de Formation, Boulevard de Waterloo 106, Bruxelles 1000, Belgium; 4 Institut Curie, Unité de Psycho-oncologie, Rue d'Ulm 26, Paris Cedex 05 75246, France

**Keywords:** training, health professionals, cancer, communication, stress, attitudes

## Abstract

There is today a wide consensus regarding the need to improve communication skills (CS) of health-care professionals (HCPs) dealing with cancer patients. Psychological training programs (PTPs) may be useful to acquire the needed CS. Testing the efficacy of PTP will allow to define their optimal content. The present study was designed to assess the impact of a PTP on HCP stress, attitudes and CS, and on HCP and patients' satisfaction with HCP communication skills in a randomised study. A total of 115 oncology nurses were randomly assigned to a 105-h PTP or to a waiting list. Stress was assessed with the Nursing Stress Scale, attitudes with a Semantic Differential Questionnaire, CS used during one simulated and one actual patient interview with the Cancer Research Campaign Workshop Evaluation Manual, and satisfaction with the nurses' CS with a questionnaire completed by the patients and the nurses. Trained (TG) and control (CG) groups were compared at baseline, after 3 months (just following training for TG) and after 6 months (3 months after the end of training for TG). Compared to controls, trained nurses reported positive changes on their stress levels (*P*⩽0.05) and on their attitudes (*P*⩽0.05). Positive training effects were found on CS used during the simulated interview: a significant increase in facilitative behaviours (open questions: *P*⩽0.001; evaluative functions: *P*⩽0.05) and a significant decrease in inhibitory behaviours (inappropriate information: *P*⩽0.01; false reassurance: *P*⩽0.05). Less positive training effects were found regarding interviews with a cancer patient: a significant increase in educated guesses (*P*⩽0.001) was noticed. No training effect was observed on nurses' satisfaction levels, but a positive training effect was found on patients' satisfaction levels (*P*⩽0.01). Although results outline PTP efficacy, they indicate the need to design PTP, amplifying the transfer of learned CS to clinical practice.

It has often been argued that working in cancer care was highly stressful for health-care professionals (HCPs) (Wilson-Barnett, 1979; Delvaux *et al*, 1988; Peetet *et al*, 1989). Moreover, HCPs' communication skills (CS) have often been reported as poor and as needing to be improved (Parle *et al*, 1997; Maguire *et al*, 1996b; Hamlin *et al*, 1999). Recommendations for the management of staff stress have therefore been given (Lederberg, 1989, pp 631–646) and the need for specific training has been raised (Razavi *et al*, 1988, 1991; Maguire, 1984; Maguire *et al*, 1990, pp 137–142; Fallowfield *et al*, 2002). In the last few decades, several educational programs have been developed, but their content and form (nature, program, length, techniques) still remain to be specified. Psychological training programs (PTPs) can be based on cognitive, emotional and/or behavioural approaches (Campbell, 1980; Shanfield, 1981; Anderson, 1982; Janetakos, 1983; Rainey *et al*, 1983; Moore, 1984; Moynihan and Outlaw, 1984; Shinn *et al*, 1984; Ziegler *et al*, 1984; Kalish, 1985; Burnard, 1991). Their effectiveness can be assessed through measuring changes in knowledge, attitudes, stress and CS (Gray-Toft, 1980a; Bensing and Sluijs, 1984; Stewart and Roter, 1990; Wilkinson *et al*, 1998, 1999; Jenkins and Fallowfield, 2002).

In the case of oncology nurses, emotional PTPs have been shown to be counter-productive, since a negative attitude shift related to job-pertinent concepts has been reported (Silberfarb and Levine, 1980). Psychological training programs with a behavioural approach could therefore be considered as being potentially more effective than programs with an emotional approach.

The efficacy of behavioural PTPs has been tested in studies, using a controlled design. Studies varied as regards length of training and outcome measures. A study on a 12-h behavioural PTP using role-playing techniques reported a positive shift in attitudes. This improvement, however, only concerned HCPs who reported the more negative attitudes at baseline. Post-training changes, moreover, were short-lived, as they were no more noticeable after 1 year (Razavi *et al*, 1988, 1991). Another study assessed the impact of a longer 24-h PTP on attitudes, on occupational stress and on CS used in simulated interviews (Razavi *et al*, 1993). This study reported a significant training effect on attitudes, especially on those related to self-concept, and on the level of occupational stress related to inadequate preparation. Limited changes were, however, found regarding CS used post-training. Moreover, 2 months after training, this study also found a loss of the training effects on stress and attitudes. The results indicated the efficacy of a 24-h PTP, and also the need to consolidate the skills acquired by regular post-training sessions. A 105-h PTP was therefore designed to increase efficacy. The primary aim of this study was to assess its efficacy in a randomised control design. The secondary aim of this study was to assess the transfer of learned CS to clinical practice.

## SUBJECTS AND METHODS

### Study design

**Figure 1:**
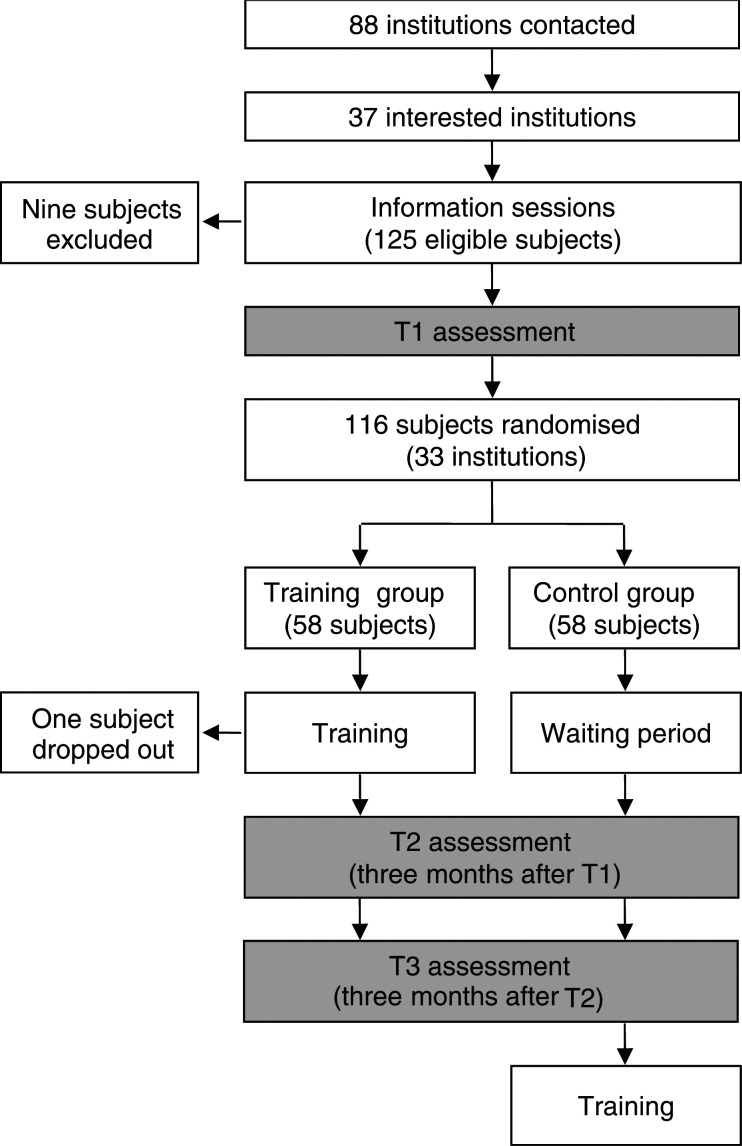
Study design and recruitment and assessment procedure.

A longitudinal randomised design was used, in which nurses wishing to participate in a PTP were allocated to a 105-h training group, or to a 6-month waiting list group (Figure 1

). After having been informed about the training and the research program, consecutive interested nurses were registered on a waiting list. Every time 20 nurses were enrolled, the nurses were randomly allocated to a training group (TG) or to a control group (CG). Training group and CG were assessed three times: at baseline (T1), 3 months after T1 (T2) and 3 months after T2 (T3). Nurses allocated to the training group (TG) were thus assessed before PTP (T1), just after PTP (T2) (3 months after T1) and 3 months later (T3). Nurses allocated to the CG were trained after having completed all their assessments. The time points for assessments are thus similar for both groups. Each subject had the same assessor throughout the study. The trainer was never involved in the assessment procedure.

### Psychological training program

The PTP included a total of 3 weeks of training (each week including 5 consecutive days): 1 week for each of the 3 consecutive months. It was designed for 10 participants. The program included 30 h of theoretical information and 75 h of role-playing exercises and of experiential exchanges. A total of 40 role-playing exercises were scheduled. Each nurse participated to in four role-playing exercises. The program is described in a detailed manual (available by writing to the authors). Aims, contents and techniques were standardised in order to allow module replication. The program was designed to decrease nurses' professional stress levels, to improve nurses' attitudes and CS.

Topics were approached according to their increased complexity. The training covered topics ranging from basic communication components in oncology, to psychosocial dimensions associated with cancer and its treatment, to coping with patients' uncertainties and distress, to detecting psychopathologic reactions to diagnosis and prognosis, and to discussing death and euthanasia. The trainer was an experienced psychologist trained in psycho-oncology and skilled in group training. To standardise the training modules, he was the only trainer of all the 12 groups (six experimental groups and six control groups).

The trainer benefited from a specific training including: information on the protocol, observation of a group in the pilot phase of the module development, participation in the elaboration of the manual describing the PTP and the role-playing exercises. Finally, the trainer was supervised regularly all over the study. During the PTP, only two theoretical presentations (pain and other physical symptoms control; psychiatric disorders) were given by a pain specialist and a psychiatrist.

### Recruitment

The PTP and research protocol was presented to the managing directors of the nursing department in 88 hospitals. These institutions were chosen on the basis of the ‘Annuaire Statistique des Hôpitaux’ established by the Minister of Public Health and Environment, according to the following criteria: having treatment facilities for cancer patients, capacity of at least 60 beds, location in the French-speaking part of Belgium or the Brussels area. A standardised information session (90 min duration) was organised in 37 interested institutions. Inclusion in the project was confirmed after an individual interview between the candidates and the study co-ordinator (inclusion visit).

To be included in the study, subjects had to be active nurses having at least a 6-months experience in cancer care, and willing to participate in a psychological training group. They understood that the training would be offered and that they had to remain available during a 6-month period for the assessment procedures.

### Assessment procedures

The assessment procedure included, at each assessment time, one simulated interview with an actress, one interview with a cancer patient and a set of questionnaires. The simulated interviews were audiotaped. The same vignette was used at T1, T2 and T3. Nurses were invited to perform a psychosocial assessment of a cancer patient with chronic pain. One actual patient interview was also audiotaped at each assessment time for each participant. Nurses were invited to perform a psychosocial assessment of a cancer patient during his first week of hospitalisation. Patients were chosen by nurses according to the following inclusion criteria: cancer patient being more than 18 years old, able to speak and read French, being free of any cognitive dysfunction and having given his written informed consent. Patients were thus different at T1, T2 and T3.

The assessment order was the following. Nurses were invited to successively complete the Nursing Stress Scale (NSS), the Semantic Differential Attitude Questionnaire and the socio-demographic questionnaire. They were then invited to participate in a 20 min audiotaped role playing with an actress (simulated interview) and in a 20 min audiotaped interview with a cancer patient (actual patient interview). After the actual patient interview, patients filled in the EORTC QLQ-C30 and each patient and each nurse filled in the Satisfaction with the Interview Assessment Questionnaire.

### Questionnaires

The NSS is originally a four-point 33-item scale describing situations that have been identified as causing stress for nurses in the performance of their duties (Gray-Toft and Anderson, 1980b). It provides a total stress score as well as scores on each of the seven subscales measuring the frequency of stress experienced by nurses in a hospital environment. The revised French version of the NSS used in this study includes 20 items. This French version provides a total score as well as scores on each of the six subscales measuring the intensity of the stress related to lack of support, inadequate preparation, professional conflicts, death and dying, caring and workload. Internal consistency coefficients were measured. Cronbach's *α* coefficients in the present sample were 0.76 for the lack of support subscale, 0.75 for the inadequate preparation subscale, 0.72 for the professional conflicts subscale, 0.68 for the death and dying subscale, 0.65 for the caring subscale and 0.78 for the workload subscale.

The Semantic Differential Attitude Questionnaire (SDAQ) was used to assess nurses' attitudes on the psychosocial aspects of cancer. The French translation (Razavi *et al*, 1993) of the SDAQ (Silberfarb and Levine, 1980) includes a list of 20 attitudes. The contrasting adjectives remain the same for each concept scored, and were chosen from the evaluative adjective scales (Osgood *et al*, 1971). The semantic differential scales are scored from 1 to 7 from the positive to the negative pole. A score is obtained for each question by adding up scores obtained on the 13 scales, and then dividing them by 13. Score 4, neutral, is allotted whenever an answer is missing. The 20 indices were grouped in five categories, reflecting attitudes about oneself, towards cancer and death, personal growth, professional relationships and occupational attitudes. For each of the five categories or factors, an average index was obtained by averaging the scores of the corresponding factor's constituent attitudes.

The EORTC QLQ-C30 is a quality of life self-evaluation for cancer patients. This questionnaire includes 30 items, and has been validated on a cancer patients' population (Aaronson *et al*, 1993). It is based on a multidimensional concept of quality of life. It includes five subscales related to functioning (physical, role, cognitive, emotional, social), three subscales related to symptoms, a quality of life global evaluation subscale and six isolated items evaluating symptoms. It was used in the present study to describe the quality of life of patients who accepted to participate in a recorded interview with the study participants at the three evaluations.

The Satisfaction with the Interview Assessment Questionnaires (SIAQ) were especially designed for the study. Nurses and patients completed their version of the questionnaire, respectively, the Nurses Satisfaction with the Interview Assessment Questionnaire (NSIAQ) and the Patient Satisfaction with the Interview Assessment Questionnaire (PSIAQ). The questionnaire is an eight-item, four-point scale ranging from 1 (not at all) to 4 (a lot). It is based on the three functions of communication (evaluation, information and support), and describes the following dimensions: satisfaction with introducing (one item), satisfaction with facilitating and listening (two items), satisfaction with informing and reassuring (three items), satisfaction with clarifying concerns (one item) and global satisfaction with the interview (one item). Internal consistency coefficients were computed for both scales. Cronbach's *α* coefficients in the present samples for the satisfaction with facilitating and listening factor were, respectively, 0.84 for the PSIAQ and 0.82 for the NSIAQ. Cronbach's *α* coefficients for the satisfaction with informing and reassuring factor were, respectively, 0.72 for the PSIAQ and 0.77 for the NSIAQ.

### Interview-rating system

The 20-min simulated and clinical interviews were transcribed and rated by trained psychologists, using the Cancer Research Campaign Workshop Evaluation Manual (CRCWEM). The CRCWEM was translated into French and adapted for the contents (Booth and Maguire, 1991). Raters were blind to the trained or untrained status of the subjects and for assessment time. The CRCWEM provides a rating for the forms, functions, blocking behaviours and psychological depth of each HCP utterance of an interview. Ratings for the forms include eight categories related to statements and different types of questions (open, directive, leading). The functions include 21 categories related to evaluative functions (psychological and general eliciting information, psychological and general clarification and checking), supportive functions (acknowledging, reassuring (true and false reassurance), negotiating and summarising), informing and advising (before or after investigation), interpretative functions (alerting to reality, confronting and educated guesses (direct or negotiated, psychological or general), introducing or concluding. The psychological depth of nurses' utterances was rated as well. The psychological depth could be absent if utterances were only about facts, or present if feelings were hinted at or mentioned explicitly.

Interviews were rated by nine intensively trained psychologists. After training, all raters had to rate the same test interview. The inter-rater reliability was measured through assessing agreement between raters on the test interview. For each individual form, function-, psychological depth- and blocking behaviour-rated agreement between all couples of raters were recorded. An inter-rater agreement rate was then calculated for each individual form, function, psychological depth and blocking behaviour, rated by summing up the number of agreements observed and dividing by the total number of possible couples. For each global category of rating, an agreement rate was then computed. There is an inter-rater agreement rate of 0.76 for the form category, 0.73 for functions, 0.73 for psychological depth levels and 0.89 for blocking behaviours. Each interview was rated by one rater. Moreover, in order to ensure a quality control and to ensure rating agreement, rating difficulties were discussed among raters to reach an agreement.

### Statistical analysis

Statistical analysis was performed using the SPSS software (SPSS, 1990). Statistical analysis of the data consisted of a comparative analysis of both groups of nurses at baseline, using parametric tests and nonparametric tests as appropriate (Student *t*-test and *χ*^2^ test). Patients' characteristics at T1 (inclusion interview), T2 (3 months later) and T3 (3 months after T2) were compared using MANOVA and *χ*^2^ tests as appropriate. Time and group-by-time changes were processed through repeated-measures analyses of variance (MANOVA). Effect sizes comparing both groups at each assessment time were computed (Cohen, 1977). All tests were two-tailed and alpha was set at 0.05. A multiple regression analysis was also performed in order to detect variables which were associated with training efficacy.

## RESULTS

### Nurses' and patients' sociodemographic data

In all, 125 nurses from 33 hospitals were potentially eligible for the study. Following the inclusion visit, nine subjects were not included in the study for the following reasons: fear of role-playing technique (*N*=1), fear of audio- and videotaped interviews (*N*=2), workload (*N*=4), lack of personal motivation (*N*=1) and other ongoing training (*N*=1). A total of 116 nurses were included in the study. One subject randomised in the TG participated in only one training week. Thus, 115 nurses were valuable for the purpose of the study. Of these, 57 subjects were randomised in the TG and 58 in the CG.

Baseline sociodemographic characteristics (age, sex, marital status, education) as well as socioprofessional characteristics (type of service, professional status, experience with cancer patients) were similar for both randomised groups (See Table 1

**Table 1:** Sociodemographic and socioprofessional data related to nurses

	**Training group (*n*=57)**			**Control Group (*n*=58)**		
	***n***		**%**	***n***		**%**
*Age*						
Mean		34.8			34.3							
Standard deviation		7.8			7.8							
Range		22–54			22–52							
						
*Sex*												
Female	51		89.5	53		91.4						
Male	6		10.5	5		8.6						
						
*Marital status*												
Single	9		15.8	19		32.8						
Separated	2		3.5	2		3.4						
Divorced	—		—	4		6.9						
Widowed	—		—	1		1.7						
Married	36		63.2	26		44.8						
With a partner	10		17.5	6		10.3						
						
*Education*												
College	6		10.5	7		12.1						
High School	49		86.0	44		75.9						
University	2		3.5	7		12.1						
						
*Professional status*												
Assistant in hospital care	—		—	2		3.4						
Certificated	20		35.1	23		39.7						
Graduated	30		52.6	28		48.3						
Manager	7		12.3	4		6.9						
No information	—		—	1		1.7						
						
*Experience with cancer patients during the last 2 years*												
1–10 patients	4		7.0	7		12.1						
>10 patients	53		93.0	51		87.9						

). The sociodemographic characteristics of cancer patients (age, sex, educational level and setting of the interview) who participated at the different assessment points (at T1, *N*=114; at T2, *N*=111; at T3, *N*=110) in the recorded interview with a HCP were also similar in the TG and the CG. Group-by-time MANOVA processing all EORTC QLQ-C30 functioning (physical, role, cognitive, emotional, social) subscales simultaneously on the one hand (*F*=1.81, NS) and all EORTC QLQ-C30 symptoms scores on the other hand (*F*=0.65, NS) are not statistically significant. The quality of life profiles of patients having been interviewed at each assessment point in the TG and the CG were thus similar.

### PTP efficacy on nurses' attitudes

The SDAQ total mean score and subscales scores (attitudes towards oneself, attitudes toward cancer and death, personal growth, professional relationships, occupational attitudes) were similar at baseline in the TG and in the CG (no statistical difference) (Table 2

**Table 2:** Post-training changes: attitudes and stress

	**T1**		**T2**		**T3**		
	**TG**	**CG**	**TG**	**CG**	**TG**	**CG**	
	**(*n*=57)**	**(*n*=58)**	**(*n*=54)**	**(*n*=58)**	**(*n*=53)**	**(*n*=58)**	
	**Mean**	**Mean**	**Mean**	**Mean**	**Mean**	**Mean**	
	**(s.d.)**	**(s.d.)**	**(s.d.)**	**(s.d.)**	**(s.d.)**	**(s.d.)**	**Group × time effect F val^a^**
*Semantic differential questionnaire (SDAQ)*							
Attitudes toward oneself	2.737	2.693	2.511	2.744	2.418	2.715	5.07**
	(0.647)	(0.700)	(0.701)	(0.735)	(0.649)	(0.778)	
Attitudes toward cancer and death	3.463	3.433	2.992	3.325	2.898	3.261	6.06**
	(0.630)	(0.774)	(0.909)	(0.854)	(0.807)	(0.834)	
Personal growth	2.274	2.387	2.194	2.381	2.169	2.402	0.50
	(0.612)	(0.705)	(0.710)	(0.626)	(0.706)	(0.672)	
Professional relationships	2.804	2.802	2.817	2.735	2.767	2.686	0.17
	(0.743)	(0.812)	(0.891)	(0.790)	(0.930)	(0.759)	
Occupational attitudes	3.144	3.117	2.851	3.112	2.731	3.070	4.91**
	(0.665)	(0.716)	(0.833)	(0.766)	(0.702)	(0.790)	
SDAQ total score	2.911	2.907	2.699	2.886	2.616	2.848	3.19*
	(0.541)	(0.576)	(0.714)	(0.647)	(0.653)	(0.677)	
							
*Nursing Stress Scale (NSS)*							
Lack of support	1.333	1.575	1.250	1.675	1.340	1.374	2.12
	(1.029)	(1.040)	(1.059)	(1.025)	(1.153)	(1.030)	
Inadequate preparation	2.597	2.449	1.425	2.173	1.222	2.144	11.21***
	(0.922)	(1.197)	(0.968)	(1.119)	(0.872)	(1.175)	
Professional conflicts	0.759	0.642	0.616	0.634	0.641	0.517	0.40
	(0.715)	(0.829)	(0.801)	(0.764)	(0.817)	(0.707)	
Death and dying	1.192	1.286	0.616	1.065	0.907	1.116	1.65
	(1.049)	(0.968)	(0.799)	(1.003)	(0.898)	(1.129)	
Caring	2.573	2.448	1.805	2.351	1.944	2.202	5.85**
	(0.821)	(0.962)	(1.016)	(0.968)	(1.045)	(1.041)	
Workload	2.035	2.224	2.013	2.408	1.810	2.121	0.21
	(1.247)	(1.176)	(1.249)	(1.211)	(1.377)	(1.278)	
NSS total score	2.129	2.046	1.700	2.039	1.621	1.812	3.07*
	(0.621)	(0.681)	(0.756)	(0.780)	(0.733)	(0.866)	

TG=training group; CG=control group;

a F-value of Fisher–Snedecor statistic (MANOVA; group × time effect) (*n* of TG=51 and *n* of CG=58) (2 ld linked to the explained variance, 106 ld linked to the residual variance);

* *P*⩽0.050;

** *P*⩽0.010;

*** *P*⩽0.001

). Group-by-time MANOVA processing all SDAQ subscales simultaneously was statistically significant (*P*⩽0.01) (Table 2). A training effect was noticeable on SDAQ total mean score (*P*⩽0.05) and on the following subscale scores: attitudes toward oneself (*P*⩽0.01), attitudes toward cancer and death (*P*⩽0.01) and occupational attitudes (*P*⩽0.01). Nurses' attitudes towards oneself, towards cancer and death and their occupational attitudes moved thus significantly more to the positive pole in the TG than in the CG.

### PTP efficacy on nurses' stress levels

Stress levels of both randomised groups measured with NSS were comparable at baseline in the TG and the CG (Table 2): NSS subscale scores (lack of support, inadequate preparation, professional conflicts, death and dying, caring, workload) and NSS total mean were similar at baseline in the TG and CG. Group-by-time MANOVA processing all NSS subscales simultaneously was statistically highly significant (*P*⩽0.001). A significant training effect was found for the total mean score (*P*⩽0.05) and for two subscale mean scores: stress related to inadequate preparation (*P*⩽0.001) and stress related to caring (*P*⩽0.01). NSS total mean score decreased significantly more in the TG. The following NSS subscales decreased significantly more in the TG: lack of support, inadequate preparation, death and dying and caring subscales.

### PTP efficacy on CS used in the simulated and in the actual patient interviews

Student's *t*-tests showed no significant differences at baseline between TG and CG for the forms, functions, psychological depths and blocking behaviours in simulated and in actual patients' interviews. Tables 3

**Table 3:** Communication skills comparison of trained and control subjects in simulated interviews

	**T1**		**T2**		**T3**			**Effect sizes (d)**		
	**TG**	**CG**	**TG**	**CG**	**TG**	**CG**				
	**(*n*=56)**	**(*n*=58)**	**(*n*=54)**	**(*n*=57)**	**(*n*=52)**	**(*n*=50)**				
	**Mean**	**Mean**	**Mean**	**Mean**	**Mean**	**Mean**				
	**(s.d.)**	**(s.d.)**	**(s.d.)**	**(s.d.)**	**(s.d.)**	**(s.d.)**	**Group × Time effect F val^a^**	**T1**	**T2**	**T3**
*Form of utterance*^b^										
Statement and response	55.66	56.96	44.65	52.53	45.29	50.82	3.10*	0.1	0.6	0.4
	(14.03)	(14.10)	(14.00)	(14.82)	(13.58)	(13.26)				
Open, open directive and screening questions	6.73	7.65	13.86	7.30	11.28	8.65	11.79***	0.1	1.0	0.3
	(5.31)	(6.61)	(7.15)	(5.31)	(8.26)	(8.17)				
Directive, leading and multiple questions	30.17	27.43	31.99	29.68	34.95	28.76	0.70	0.2	0.2	0.5
	(13.99)	(12.29)	(11.41)	(12.36)	(12.07)	(12.53)				

*Function of utterance*^b^										
Introducing, concluding	2.39	2.37	2.58	2.32	2.41	2.32	0.38	0.0	0.2	0.1
	(1.31)	(1.82)	(1.41)	(1.71)	(1.30)	(1.72)				
Eliciting information, clarification, and checking	32.67	32.01	41.43	32.68	42.24	33.67	4.37*	0.0	0.7	0.6
	(14.10)	(13.85)	(13.06)	(13.25)	(13.39)	(14.51)				
Information after investigation (appropriate)	8.85	6.74	7.93	9.95	10.70	9.87	1.20	0.3	0.3	0.1
	(7.63)	(6.44)	(5.89)	(8.51)	(6.33)	(8.58)				
Information before investigation (inappropriate)	15.83	16.53	5.42	11.14	4.39	12.67	5.57**	0.1	0.7	1.0
	(9.57)	(12.94)	(6.23)	(9.21)	(3.87)	(11.58)				
Acknowledgement, empathy, negotiating, summary	18.10	19.41	21.75	18.59	21.32	16.05	2.74	0.1	0.3	0.4
	(10.39)	(12.49)	(10.25)	(12.25)	(13.19)	(10.78)				
Reassurance	1.23	0.81	0.81	1.21	0.88	0.62	1.94	0.2	0.2	0.2
	(1.91)	(1.62)	(1.54)	(1.96)	(1.63)	(1.62)				
False or premature reassurance	6.18	6.04	1.85	5.25	2.14	4.98	3.52*	0.0	0.7	0.6
	(4.94)	(6.00)	(3.19)	(5.89)	(2.68)	(5.69)				
Educated guesses, alerting to reality, confronting	7.29	8.13	8.74	8.36	7.44	8.06	0.37	0.1	0.1	0.1
	(5.71)	(5.83)	(5.72)	(5.27)	(4.59)	(6.45)				

*Psychological depth of the interview*^*b*^										
About facts only	55.92	56.04	36.83	47.93	37.96	42.94	1.85	0.0	0.6	0.3
	(24.78)	(21.78)	(16.36)	(18.17)	(16.03)	(18.01)				
Feelings hinted at or mentionned explicitly	36.70	36.07	53.75	41.59	53.59	45.29	2.70	0.0	0.7	0.5
	(23.30)	(20.75)	(16.01)	(18.70)	(16.29)	(18.82)				

*Blocking behaviours*^b^										
No blocking	65.52	62.47	73.84	62.81	74.63	60.57	5.17**	0.2	0.7	0.9
	(17.12)	(15.71)	(14.11)	(15.98)	(11.60)	(17.80)				
Blocking, repetition and repetition as blocking	27.10	29.64	16.73	26.72	16.95	27.62	3.77*	0.2	0.7	0.8
	(15.64)	(14.09)	(12.49)	(16.05)	(11.35)	(16.32)				

TG=training group; CG=control group.

a F-value of Fischer–Snedecor statistic (Manova; group × time effect) (*n* of TG=51 and *n* of CG=50) (2 ld linked to the explained variance, 98 ld linked to the residual variance).

b Percentages of unrated utterances (inaudible, incomplete or unclassifiable) are not reported in the table; these percentages may be calculated for each category of rating.

* *P*⩽0.050;

** *P*⩽0.010;

*** *P*⩽0.001.

and 4

**Table 4:** Communication skills comparison of trained and control subjects in actual patient interviews

	**T1**		**T2**		**T3**			**Effect sizes (d)**		
	**TG**	**CG**	**TG**	**CG**	**TG**	**CG**				
	**(*n*=53)**	**(*n*=54)**	**(*n*=53)**	**(*n*=54)**	**(*n*=51)**	**(*n*=54)**				
	**Mean**	**Mean**	**Mean**	**Mean**	**Mean**	**Mean**				
	**(s.d.)**	**(s.d.)**	**(s.d.)**	**(s.d.)**	**(s.d.)**	**(s.d.)**	**Group × time effect F val^a^**	**T1**	**T2**	**T3**
*Form of utterance*^b^										
Statement and response	49.96	51.17	52.58	49.99	49.48	50.82	0.18	0.1	0.2	0.1
	(15.74)	(14.48)	(14.54)	(15.65)	(10.25)	(13.74)				
Open, open directive and screening questions	5.12	6.82	5.53	5.73	5.55	4.71	1.95	0.4	0.0	0.2
	(4.38)	(5.16)	(3.75)	(5.34)	(4.63)	(4.18)				
Directive, leading and multiple questions	32.31	29.13	30.91	30.94	33.70	33.78	0.26	0.2	0.0	0.0
	(14.41)	(11.23)	(12.28)	(14.14)	(10.15)	(12.41)				

*Function of utterance*^b^										
Introducing, concluding	1.53	2.15	1.76	1.63	1.53	1.41	0.78	0.2	0.1	0.1
	(2.58)	(3.68)	(1.63)	(2.14)	(1.48)	(1.73)				
Eliciting information, clarification and checking	36.46	34.52	35.50	36.18	37.93	37.73	0.02	0.1	0.0	0.0
	(15.10)	(13.25)	(13.28)	(15.00)	(11.59)	(13.93)				
Information after investigation (appropriate)	7.22	6.91	5.64	6.03	5.62	6.48	1.53	0.0	0.1	0.1
	(7.62)	(7.46)	(7.51)	(5.53)	(4.22)	(7.45)				
Information before investigation (inappropriate)	1.41	1.32	2.30	2.13	1.09	1.00	0.40	0.0	0.0	0.0
	(2.49)	(2.63)	(6.68)	(4.55)	(1.92)	(2.75)				
Acknowledgement, empathy, negotiating, summary	37.05	36.96	39.18	37.66	39.30	40.31	0.10	0.0	0.1	0.1
	(15.68)	(14.64)	(14.44)	(13.71)	(12.78)	(15.17)				
Reassurance	0.37	0.59	0.25	0.41	0.14	0.25	0.03	0.2	0.2	0.2
	(0.91)	(1.53)	(0.53)	(0.88)	(0.43)	(0.69)				
False or premature reassurance	0.49	0.64	0.54	0.63	0.56	0.64	0.35	0.1	0.1	0.1
	(0.91)	(1.36)	(1.03)	(0.22)	(1.57)	(1.12)				
Educated guesses, alerting to reality, confronting	2.86	4.04	3.85	1.10	2.56	1.50	5.32**	0.3	0.5	0.4
	(3.72)	(4.14)	(4.45)	(2.70)	(2.72)	(2.19)				

*Psychological depth of the interview*^b^										
About facts only	63.59	63.48	70.66	72.34	67.19	76.71	3.00	0.1	0.1	0.6
	(20.90)	(18.93)	(16.26)	(19.37)	(17.86)	(16.51)				
Feelings hinted at or mentionned explicitly	21.95	24.01	18.76	14.58	21.69	12.72	2.70	0.1	0.3	0.6
	(18.32)	(16.53)	(19.90)	(15.62)	(17.43)	(13.75)				

*Blocking behaviours*^b^										
No blocking	83.47	82.40	84.74	81.11	85.15	85.44	0.91	0.1	0.3	0.0
	(10.71)	(10.21)	(9.69)	(13.45)	(9.62)	(8.47)				
Blocking, repetition and repetition as blocking	4.07	5.17	4.70	5.81	3.76	3.99	0.37	0.3	0.2	0.1
	(3.76)	(4.64)	(4.73)	(6.38)	(4.48)	(3.87)				

TG=training group; CG: Control Group;

a F-value of Fischer–Snedecor statistic (Manova; group × time effect) (*n* of TG=51 and *n* of CG=50) (2 ld linked to the explained variance 98 ld linked to the residual variance).

b Percentages of unrated utterances (inaudible, incomplete or unclassifiable) are not reported in the table; these percentages may be calculated for each category of rating.

^*^P⩽0.050;

** *P*⩽0.010.

report the means and standard deviations of percentages of nurses' CS together with MANOVA F and P-values and effect sizes. As shown in Table 3, several significant MANOVA group-by-time changes were observed in simulated interviews. Over time, nurses in the TG, compared with nurses in the CG, made less statements (*P*⩽0.05) and asked more open, open directive and screening questions (*P*⩽0.001). They elicited, clarified and checked more information (*P*⩽0.05), gave less inappropriate information (*P*⩽0.01) and made less false reassurances (*P*⩽0.05). Blocking behaviours decreased significantly in the TG (no blocking behaviours: *P*⩽0.01). No group-by-time effect was found for the psychological depth of the utterances.

As shown in Table 4, few significant MANOVA group-by-time changes in nurses' CS were found in actual patient interviews. Nurses in the TG used more educated guesses, alerting to reality and confronting at T2 and T3 compared to the CG (*P*⩽0.01). No group-by-time effects were found for the psychological depth of the utterances and for blocking behaviours.

### PTP efficacy on nurses' and patients' satisfaction with the interview

Group-by-time MANOVA processing all NSIAQ subscales simultaneously was not statistically significant (*P*⩾0.10). Responses of nurses about the interview (Table 5

**Table 5:** Nurse and patient satisfaction with the interview assessment questionnaire

	**T1**		**T2**		**T3**		
	**TG**	**CG**	**TG**	**CG**	**TG**	**CG**	
	**Mean**	**Mean**	**Mean**	**Mean**	**Mean**	**Mean**	
	**(s.d.)**	**(s.d.)**	**(s.d.)**	**(s.d.)**	**(s.d.)**	**(s.d.)**	**Group × time effect F val^a^**
*Nurse satisfaction (NSIAQ)*^a^	(*n*=55)	(*n*=57)	(*n*=52)	(*n*=57)	(*n*=52)	(*n*=58)	
Introduction	2.96	2.98	3.00	3.19	3.08	3.09	1.39
	(0.77)	(0.64)	(0.75)	(0.72)	(0.68)	(0.73)	
Clarification of the preoccupations	2.47	2.49	2.54	2.48	2.63	2.40	0.70
	(0.74)	(0.71)	(0.64)	(0.71)	(0.72)	(0.75)	
Information and support	1.91	1.96	2.00	1.97	1.91	1.90	0.33
	(0.67)	(0.54)	(0.60)	(0.64)	(0.57)	(0.58)	
Facilitation and listening	3.26	3.33	3.47	3.22	3.36	3.16	4.16*
	(0.58)	(0.49)	(0.52)	(0.57)	(0.57)	(0.61)	
Global satisfaction	2.71	2.71	2.65	2.77	2.69	2.64	0.31
	(0.76)	(0.71)	(0.65)	(0.71)	(0.70)	(0.87)	

*Patient satisfaction (PSIAQ)*^b^	(*n*=57)	(*n*=57)	(*n*=54)	(*n*=57)	(*n*=52)	(*n*=58)	
Introduction	3.63	3.44	3.31	3.39	3.64	3.25	2.97
	(0.56)	(0.82)	(0.87)	(0.82)	(0.60)	(0.79)	
Clarification of the preoccupations	2.95	3.21	3.06	2.96	3.24	2.80	3.92*
	(1.03)	(0.97)	(1.01)	(1.05)	(0.99)	(0.98)	
Information and support	2.64	2.98	2.75	2.68	2.86	2.55	4.82**
	(0.92)	(0.91)	(0.99)	(0.94)	(0.90)	(0.89)	
Facilitation and listening	3.81	3.75	3.82	3.82	3.76	3.84	0.49
	(0.38)	(0.56)	(0.38)	(0.35)	(0.65)	(0.33)	
Global satisfaction	3.67	3.63	3.59	3.70	3.75	3.62	1.48
	(0.51)	(0.70)	(0.57)	(0.46)	(0.48)	(0.52)	

a F-value of Fisher–Snedecor statistic (MANOVA; group × time effect) (*n* of TG=48 and *n* of CG=56) (2 ld linked to the explained variance, 101 ld linked to the residual variance).

b F-value of Fisher–Snedecor statistic (MANOVA; group × time effect) (*n* of TG=52 and *n* of CG=56) (2 ld linked to the explained variance, 105 ld linked to the residual variance).

* *P*⩽0.050;

** *P*⩽0.010.

) showed a training effect on ‘facilitation and listening’ (*P*⩽0.05).

Group-by-time MANOVA processing all PSIAQ subscales simultaneously was not statistically significant (*P*⩾0.10). Responses of patients about the interview (Table 5) showed a significant training effect: patients perceived an improvement of HCP skills related to ‘clarification of the preoccupations’ (*P*⩽0.05) and ‘information and support’ (*P*⩽0.01).

### Predictors of PTP efficacy on evaluative functions

One model was tested with multivariate analysis on evaluative function improvements in the TG between T1 and T2 during the simulated interview. The model provided a significant multiple correlation (multiple *R*=0.762; *P*⩽0.001), including the following variables: evaluative skills before training (beta=−0.566; *P*⩽0.001), stress before training (beta=0.312; *P*⩽0.01), number of cancer patients seen since 2 years (beta=0.258; *P*⩽0.05), clinical experience in years (beta=0.188; *P*⩽0.10).

## DISCUSSION AND CONCLUSION

As it was expected, a relatively small number of potential hospitals agreed to participate in the study (37 of the 88 hospitals initially approached). The reason could be linked with the fact that many hospitals were unwilling to free up the requisite nursing staff time (105 h) for such training. It should be underlined, however, that a majority of nurses who were given the opportunity to attend the program agreed to participate.

This randomised study assessing a 105-h PTP gives an in-depth understanding of its efficacy. Results confirm the training impact on professional stress and attitudes. Trained nurses felt less stressed in general, less stressed by giving ‘painful’ or ‘ineffective’ caring, and better prepared to provide emotional support to patients and their family. Moreover, nurses' attitudes towards cancer and death, towards oneself and occupational attitudes moved to the positive pole. It should be underlined that, compared with a previous study assessing the effectiveness of a 24-h PTP using the same design and tools (Razavi *et al*, 1993), this longer training program led to more improvements. Improvements which were contrary to what was observed with the shorter PTP lasted overtime.

Most importantly for the focus of this study, this 105-h PTP showed significant improvements in CS both in simulated and in actual patient interviews. Most improvements were observed in simulated interviews. Just after training and 3 months later, facilitative behaviours (open questions, evaluative functions) significantly increased and inhibitory behaviours (statements and responses information without investigation, false reassurance and blocking) significantly decreased. In actual patient interviews, trained nurses only used more educated guesses, alerting to reality and confronting utterances. The relatively small number of CS improvements observed after training in actual patient interviews may be explained by the fact that nurses may approach simulated interviews like an exercise directly related to the role-playing exercises they performed during training. Lack of improvement observed after training in actual patient interviews could also be in part explained by the heterogeneity of actual patient interviews that could not allow changes to be observed. Finally, the lack of transfer of learning to the clinical setting may also be related to the fact that training does not alleviate some stresses (lack of support, professional conflicts and workload), which could reduce motivation to use the acquired CS in the clinical setting. After training, nurses are certainly more conscious of their inhibitory behaviours. Knowledge about basic CS is acquired, but not fully applied in the context of actual patient interviews. Comparison of these results with other studies assessing shorter PTP efficacy showed, however, that a longer training module was associated with more beneficial effects on CS and with more stability of these effects over time (Razavi *et al*, 1993; Maguire *et al*, 1996a).

Results of this 105-h PTP on nurses and patients satisfaction should be stressed. Despite this small number of facilitative CS changes observed in actual patient interviews, patients interacting with trained nurses reported a higher level of satisfaction with some dimensions of nurses CS (introduction, concern clarification, information and reassurance). This result is interesting as few studies have reported an impact of training on patients' satisfaction. As it is often observed in studies, patients' levels of satisfaction with their nurses were at ceiling already at baseline. The construction of the scale on the basis of the three functions of communication (evaluation, information and support) allowed, however, to highlight changes in dimensions of patients' satisfaction that are often not recorded. Nurses finally also reported a higher satisfaction with their listening attitudes.

This study finally sheds some light on factors associated with the amplitude of training effects. Changes in the number of evaluative functions performed during a simulated interview were chosen as a dependant variable. Regression analysis showed that nurses who benefited most of the training had poorer CS at baseline, more working experience with cancer patients, and reported more professional stress. It can be hypothesised that experienced nurses with poor CS may have developped professional stress that may have induced a higher motivation for training, and may explain that they benefit most of the training.

This randomised study assessing PTP for HCPs dealing with cancer care is a step in the understanding of its efficacy. These results emphasise the potential value of organising regular feedback and supervision at the workplace after a training program. They also provide evidence of the added value of a 105-h program in terms of the number of changes observed and in terms of the lasting effect of the changes. Despite efficacy reported here, a number of questions remain unresolved and need to be assessed in controlled studies: effects of monodisciplinary *vs* multidisciplinary workshops, effects of more interactive training techniques, effects of training techniques teaching a broader range of CS, effects of modelling and effects of regular feedback on performed clinical skills in every day work. The usefulness of consolidation workshops, supervision workshops and coaching procedures in order to transfer learned CS to the clinical setting should be assessed for nurses, as it has been done recently for physicians (Razavi *et al*, 2003). The development of research focusing on training efficacy in terms of patients' benefits (Betz Brown *et al*, 1999) and cost utility is also needed, as this study supports the idea that improved HCP CS is directly related to patient increased satisfaction with care.
